# NiS_*x*_/NiO/MoS_2_ heterostructure for enhanced hydrogen evolution in acidic media and supercapacitor electrode applications

**DOI:** 10.1039/d5ra06931a

**Published:** 2026-01-14

**Authors:** Sumaiya Saleem, Muhammad Salman, Ilham Noor, Baseena Sardar, Majid Khan

**Affiliations:** a Department of Physics, Abdul Wali Khan University Mardan Mardan 23200 Pakistan majidkhan@awkum.edu.pk; b Department of Physics, University of Malakand Chakdara 18800 Pakistan

## Abstract

This study demonstrates the synthesis and characterization of a NiS_*x*_/NiO/MoS_2_ (*x* = 1, 2) heterostructure using a hydrothermal method, demonstrating superior performance for the hydrogen evolution reaction (HER) in 1 M H_2_SO_4_. X-ray diffraction (XRD) confirmed the presence of cubic NiO, mixed NiS and NiS_2_ phases, and hexagonal MoS_2_, while scanning electron microscopy (SEM) revealed an interconnected nanosheet morphology with Ni, S, Mo, and O elements verified by energy-dispersive X-ray (EDX) spectroscopy. Fourier-transform infrared (FTIR) spectroscopy identified Ni–O, Ni–S, and Mo–S bonds, and UV-visible spectroscopy indicated a reduced bandgap of 2.81 eV for the composite. The composite achieved a low overpotential of 166 mV at 10 mA cm^−2^, a Tafel slope of 55 mV dec^−1^, and a charge transfer resistance of 52 Ω, outperforming individual NiO (582 mV, 91 mV dec^−1^, 266 Ω) and NiS_*x*_ (357 mV, 72 mV dec^−1^, 170 Ω). Additionally, the composite exhibited a high specific capacitance of 428.24 F g^−1^ at 2 A g^−1^ and an electrochemical surface area (ECSA) of 0.7 mF cm^−2^, highlighting its potential as a dual-functional material for HER and supercapacitor applications. These results underscore the effectiveness of synergistic interactions in enhancing catalytic activity and charge storage capacity, offering a promising pathway for sustainable energy technologies.

## Abstract 2

A NiO/NiS_*x*_/MoS_2_ nanocomposite enables boosted HER performance and superior specific capacitance in a bifunctional energy material.

## Graphical abstract

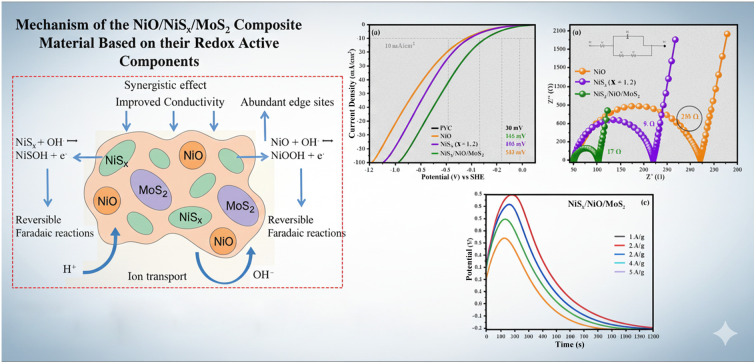

## 1. Introduction

The rapid expansion of the global economy and urbanization, coupled with significant improvements in living standards, has led to a surge in energy demand and exacerbated environmental challenges, including the depletion of fossil fuels and escalating greenhouse gas emissions.^1^ These issues have underscored the urgent need for sustainable and clean energy solutions. Hydrogen, as the lightest and most abundant element in the universe, has emerged as a pivotal energy carrier due to its zero carbon emissions, high energy density, and recyclability. Although primarily found in compound forms such as water and hydrocarbons on Earth, extracting hydrogen from these sources necessitates energy-efficient and sustainable methods.^2^

Among various hydrogen production techniques, water electrolysis powered by renewable energy sources like solar or wind has gained prominence as a promising approach. This process involves the electrocatalytic splitting of water into hydrogen and oxygen, yielding high-purity hydrogen without harmful by-products. When hydrogen is subsequently utilized in fuel cells, it reacts with oxygen to generate electricity, with water as the only by-product, offering a clean and sustainable energy solution.^3,4^ Electrochemical water splitting, particularly when driven by renewable electricity, stands out as one of the most efficient and scalable methods for sustainable high-purity hydrogen production. The process hinges on two key half-cell reactions: the hydrogen evolution reaction (HER) at the cathode and the oxygen evolution reaction (OER) at the anode.^2,5^ The efficiency of these reactions is critically dependent on the electrocatalyst, which plays a vital role in reducing the activation energy required for the reactions. An ideal HER catalyst must exhibit high catalytic activity, low overpotential, and optimal hydrogen adsorption energy.^6^ In parallel with hydrogen technologies, other electrochemical energy storage devices such as lithium-ion batteries, sodium-ion batteries, supercapacitors, and hybrid capacitive systems have been extensively studied due to their complementary roles in addressing the global energy challenge. Lithium-ion and sodium-ion batteries offer high energy density and are widely used for grid storage and portable electronics. Supercapacitors, especially those based on electrochemical double-layer capacitance (EDLC) and pseudocapacitance, provide ultrafast charge–discharge rates, high power density, and long cycle life, making them suitable for high-power applications and regenerative energy systems. Hybrid energy storage systems combine the advantages of both technologies, delivering both high energy and power densities with enhanced cycling stability.^7–9^ These diverse technologies collectively underscore the importance of developing advanced materials for both energy conversion and storage applications. While platinum (Pt) and Pt-based electrocatalysts are renowned for their superior performance, their high cost, limited availability, and susceptibility to degradation pose significant barriers to large-scale commercialization.^4,10^ Consequently, extensive research has been directed toward developing alternative electrocatalysts for HER. Various materials have been explored, including metal oxides, nitrides, carbides, sulfides, phosphides, alloys, transition-metal dichalcogenides (TMDs), transition-metal oxides (TMOs), and carbon-based materials.^6,11–19^ Among these transition-metal sulfides (TMSs), particularly molybdenum disulfide (MoS_2_), have garnered significant attention due to their earth abundance, tunable electronic properties, and high electrocatalytic activity.^20^

MoS_2_, a member of the TMD family, possesses unique properties such as a layered structure, high surface area, and mechanical flexibility, making it a promising candidate for HER.^6,21^ However, its catalytic efficiency is constrained by low electrical conductivity and weak hydrogen adsorption capability. In its natural 2H phase, only the edge sites of MoS_2_ are catalytically active, while the basal planes remain inert.^6,22–26^ To overcome these limitations, recent advancements have focused on modifying MoS_2_ through strategies such as defect engineering, doping, and the formation of heterostructures or composites.^25–28^ Notably, doping MoS_2_ with nickel has been shown to activate the basal planes, significantly enhancing its HER performance. Studies have demonstrated that nickel single atoms can be incorporated into MoS_2_ nanosheets, reducing the hydrogen adsorption free energy on the basal planes to levels comparable to the active edge sites.^25^ Such modifications highlight the potential of MoS_2_-based composites in achieving high catalytic activity.

Nickel-based catalysts, particularly nickel sulfides, have also gained considerable attention for their excellent HER performance across different pH conditions. Nickel sulfides such as NiS and NiS_2_ offer advantages like simple synthesis, low cost, and high catalytic activity. These materials have been studied in various nanostructured forms and have shown remarkable HER performance, especially in acidic media.^29–37^ Furthermore, the formation of composites or heterostructures involving MoS_2_ and silver-based or cobalt-based, or nickel-based materials has proven to be a successful strategy for enhancing catalytic performance.^38–40^ For instance, NiS_2_/CoS_2_/MoS_2_ composites have been synthesized to leverage the synergistic effects of these materials, resulting in improved HER activity.^25^ These composites benefit from the enhanced electrical conductivity and increased active sites provided by nickel-based components, which complement the catalytic properties of MoS_2_.

In this study, we synthesized NiS_*x*_, NiO, and NiS_*x*_/NiO/MoS_2_ (*x* = 1, 2) heterostructure and evaluated their HER performance in an acidic medium. Our results demonstrate that the ternary composite exhibits enhanced catalytic properties compared to the individual components, highlighting its potential as an efficient electrocatalyst for hydrogen evolution. This work contributes to the ongoing efforts to develop cost-effective and high-performance electrocatalysts for sustainable hydrogen production, addressing the critical need for clean energy solutions in the face of global environmental challenges.

## 2. Materials and methods

### 2.1. Materials

All chemicals used in this study were of analytical grade and used without further purification. Deionized water (H_2_O), ethanol (C_2_H_5_OH), methanol (CH_3_OH), Triton X-100, sodium hydroxide (NaOH), nickel chloride (NiCl_2_), and thiourea (CS(NH_2_)_2_) were procured from Sigma-Aldrich (China) with a purity of 99.9%. Sodium molybdate dihydrate (NaMoO_4_·2H_2_O) was also obtained from Sigma-Aldrich. Polyvinylpyrrolidone (PVP, (C_6_H_9_NO)_*n*_) was purchased from Sigma-Aldrich for use as a binder in electrochemical experiments. All solutions were prepared using deionized water and analytical-grade solvents to ensure high purity and reproducibility.

### 2.2. Synthesis of nickel oxide (NiO)

The hydrothermal method allows better control over structure and morphology, which can enhance electrochemical or catalytic performance. The controlled morphology, such as nanosheets, nanorods, or porous structures, provides a larger surface area and abundant active sites, which facilitate enhanced ion/electron transport and improve redox kinetics. Furthermore, well-defined structures with optimized porosity enhance electrolyte accessibility and reduce diffusion paths, thereby improving electrochemical performance. Similarly, in catalytic applications, tailored morphology and crystal structures expose more reactive facets, which reduce charge transfer resistance and thereby contribute to higher catalytic efficiency. Nickel oxide (NiO) was synthesized *via* a hydrothermal method followed by annealing. A 0.2 M solution of nickel chloride (NiCl_2_) was prepared by dissolving the appropriate amount in an 80 mL mixture of deionized water and ethylene glycol (50 : 50 by weight) under continuous magnetic stirring at room temperature. In a separate beaker, a 0.4 M sodium hydroxide (NaOH) solution was prepared in an identical 80 mL mixture of deionized water and ethylene glycol, stirred until fully dissolved. The NaOH solution was then added dropwise to the NiCl_2_ solution using a pipette, with continuous stirring for 30 minutes to ensure complete mixing. The resulting mixture was transferred to a 200 mL Teflon-lined stainless steel autoclave and heated in a preheated oven at 180 °C for 12 hours. After cooling to room temperature, the precipitate was collected and washed multiple times with deionized water and ethanol through centrifugation and sonication to remove residual impurities. The washed precipitate was dried at 60 °C for 12 hours, yielding a light greenish powder identified as nickel hydroxide (Ni(OH)_2_). This powder was annealed at 400 °C for 3 hours in a furnace under ambient conditions, converting it to a black NiO powder.

### 2.3. Synthesis of nickel sulfide (NiS)

Nickel sulfide (NiS) was synthesized using the Ni(OH)_2_ powder obtained prior to annealing in the NiO synthesis. A 0.1 M solution of Ni(OH)_2_ was prepared by dispersing the powder in an 80 mL mixture of deionized water and ethylene glycol (EG) 50 : 50 by weight under magnetic stirring (EG generally plays a critical role in the experimental route as a solvent, reducing agent, and morphology-directing medium). In a separate beaker, a 0.1 M solution of thiourea (CH_4_N_2_S) was dissolved in an identical 80 mL mixture and stirred until fully dissolved. The thiourea solution was then added to the Ni(OH)_2_ solution, and the mixture was stirred for an additional 30 minutes to ensure homogeneity. The combined solution was transferred to a 200 mL Teflon-lined stainless steel autoclave and heated at 180 °C for 18 hours in a preheated oven. After cooling to room temperature, the resulting precipitate was washed multiple times with ethanol and deionized water using centrifugation and sonication to remove unreacted reagents and by-products. The washed product was dried at 60 °C overnight in a drying oven to eliminate residual solvents. The dried powder was then annealed in a vacuum furnace at 400 °C for 3 hours to yield NiS.

### 2.4. Synthesis of NiS_*x*_/NiO/MoS_2_ (*x* = 1, 2) heterostructure

The NiS_*x*_/NiO/MoS_2_ (*x* = 1, 2) heterostructure was synthesized *via* a hydrothermal method. Two separate 80 mL mixtures of deionized water and ethylene glycol (50 : 50 by weight) were prepared in two beakers. In the first beaker, 0.276 M Ni(OH)_2_, prepared as described in Section 2.2, was dispersed under magnetic stirring. Half of a 0.207 M thiourea (CH_4_N_2_S) solution was added to this beaker and stirred until fully dissolved. In the second beaker, 0.138 M sodium molybdate dihydrate (NaMoO_4_·2H_2_O) was dissolved in the same solvent mixture. The remaining half of the 0.207 M thiourea solution was added to the second beaker and stirred until fully dissolved. Subsequently, 0.5 mL of Triton X-100 was added to the Ni(OH)_2_ and thiourea-containing solution as a surfactant/stabilizer to enhance dispersion and stirred for 25 minutes. The solution from the second beaker (containing NaMoO_4_·2H_2_O and thiourea) was then added to the first beaker, and the combined mixture was stirred for an additional 30 minutes to ensure thorough mixing. The final solution was transferred to a 200 mL Teflon-lined stainless steel autoclave and heated at 180 °C for 18 hours in a preheated oven. After cooling to room temperature, the precipitate was washed multiple times with deionized water and ethanol through centrifugation and sonication to remove impurities. The washed product was dried at 60 °C overnight to yield the NiS_*x*_/NiO/MoS_2_ (*x* = 1, 2) heterostructure (Fig. S1).

### 2.5. Physical characterization

The structural properties and phase purity of the synthesized samples were analysed using X-ray diffraction (XRD) with a D8 ADVANCE instrument (Bruker) over a 2-theta range of 20°–80°. Surface morphology was examined using scanning electron microscopy (SEM, Hitachi SU8200), and elemental composition was determined *via* energy-dispersive X-ray (EDX) spectroscopy coupled with the SEM system. Fourier-transform infrared (FTIR) spectroscopy was performed using an InfraLUM FT-08 spectrometer to investigate the functional groups and bonding characteristics, with spectra collected in the range of 400–4000 cm^−1^. Optical properties in the ultraviolet and visible regions were evaluated using a UV-visible spectrophotometer (Peak Instrument C-7200) at room temperature.

### 2.6. Electrochemical characterization

Electrochemical properties were investigated using a three-electrode system with a 1 M sulfuric acid (H_2_SO_4_) solution as the electrolyte. A glassy carbon electrode served as the working electrode, an Ag/AgCl electrode as the reference electrode, and a graphite rod electrode as the counter electrode. Polyvinylpyrrolidone (PVP, (C_6_H_9_NO)_*n*_), purchased from Sigma-Aldrich, was used as a binder for electrode preparation. Electrochemical measurements, including cyclic voltammetry (CV), *iR*-corrected linear sweep voltammetry (LSV), and electrochemical impedance spectroscopy (EIS), were conducted using an AutoLab Metrohm instrument (serial number AUT52640). To prepare the working electrode, 1 mg of the sample (NiO, NiS, or NiS_*x*_/NiO/MoS_2_ (*x* = 1, 2) heterostructure) was dispersed in 80 µL of PVP binder and sonicated for 30 minutes to ensure homogeneity. Approximately 5 µL of the resulting ink was deposited onto the glassy carbon electrode and dried at 55 °C for 5 minutes in a drying oven to ensure proper adhesion of the sample to the electrode surface.

## 3. Results and discussion

### 3.1. Structural and phase analysis by X-ray diffraction (XRD)

**Fig. 1:**
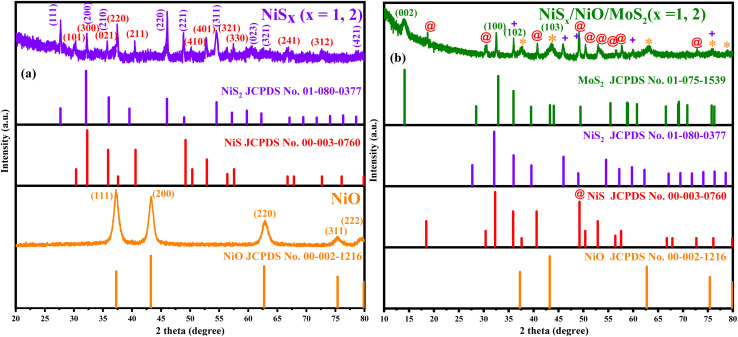
XRD pattern along with reference cards (a) NiO and NiS_*x*_ (*x* = 1, 2), and (b) NiS_*x*_/NiO/MoS_2_ (*x* = 1, 2) heterostructure.

To investigate the crystalline structure and phase composition of the synthesized materials, X-ray diffraction (XRD) analysis was performed over a 2-theta range of 20° to 80° for NiO and NiS_*x*_ (*x* = 1, 2), and 10°–80° for the NiS_*x*_/NiO/MoS_2_ composite, as shown in Fig. 1. The XRD pattern of NiO (Fig. 1(a)) exhibits distinct diffraction peaks at 2-theta values of 37°, 43°, 63°, 75°, and 80°, which align closely with the JCPDS card no. 002-1216 for cubic NiO with the *Fm*3*m* space group. These peaks correspond to the (111), (200), (220), (311), and (222) crystallographic planes of bunsenite NiO, confirming the successful formation of phase-pure NiO with a cubic structure. The sharp and intense peaks indicate high crystallinity, consistent with the annealing process at 400 °C, which enhances lattice ordering.^6^ For the NiS_*x*_ sample (Fig. 1(a)), the XRD pattern reveals a mixed-phase composition of NiS and NiS_2_. Peaks at 30°, 32°, 36°, 38°, 41°, 50°, 53°, 58°, 67°, 70°, and 73° correspond to the rhombohedral NiS phase (JCPDS card no. 003-0760, space group *R*3*m*), matching the (101), (300), (021), (220), (211), (410), (401), (321), (330), (241), and (312) planes of millerite NiS, respectively. Additionally, peaks at 28°, 32°, 36°, 46°, 49°, 55°, 60°, 62°, and 79° align with the cubic NiS_2_ phase (JCPDS card no. 01-080-0377, space group *Pa*3), corresponding to the (111), (200), (210), (220), (221), (311), (023), (321), and (421) planes. The peaks at 32° and 36° are common to both NiS and NiS_2_, indicating the coexistence of both phases. This mixed-phase formation is attributed to the hydrothermal synthesis conditions and the sulfur content from thiourea, which facilitates the formation of both NiS and NiS_2_.^30^

The XRD pattern of the NiS_*x*_/NiO/MoS_2_ (*x* = 1, 2) heterostructure (Fig. 1(b)) exhibits a characteristic peak at 2-theta less than 15°, corresponding to the (002) plane of hexagonal MoS_2_ (JCPDS card no. 75-1539), confirming the presence of MoS_2_ in the composite. Additional peaks align with those observed for NiO, NiS, and NiS_2_, indicating the successful integration of all three components in the composite. However, a notable decrease in the intensity of some NiS and NiO peaks, along with the disappearance of minor peaks, suggests potential overlap or suppression by the stronger NiS phases or the incorporation of MoS_2_. This could also be due to a reduction in Ni^2+^ concentration, which may weaken the diffraction signals of NiS and NiO, as reported in previous studies.^41^ The presence of all expected phases confirms the successful formation of the NiS_*x*_/NiO/MoS_2_ (*x* = 1, 2) heterostructure, with the MoS_2_ contributing to the layered structure of the composite, potentially enhancing its catalytic properties.

### 3.2. Fourier-transform infrared (FTIR) spectroscopy analysis

**Fig. 2:**
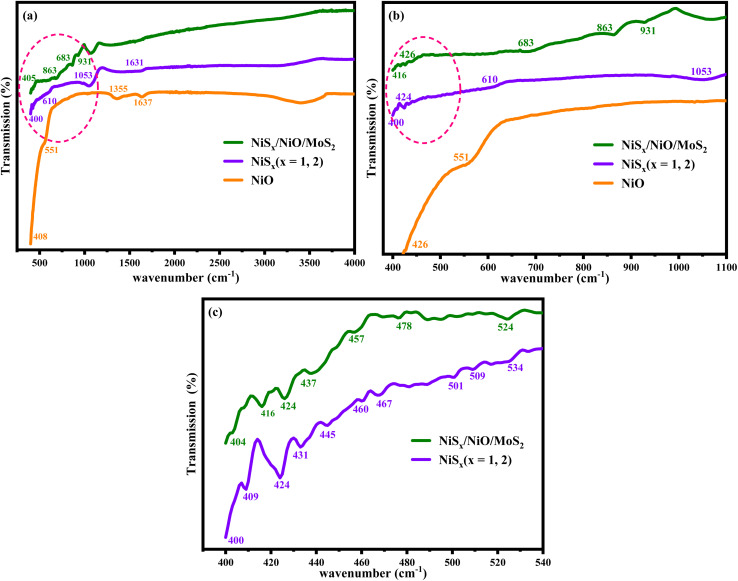
FTIR spectra of NiS_*x*_/NiO/MoS_2_ in comparison with NiO and NiS_*x*_ (*x* = 1, 2) at different spectral ranges (a) 400–4000 cm^−1^, (b) 400–1100 cm^−1^, and (c) NiS_*x*_/NiO/MoS_2_ (*x* = 1, 2) heterostructure in comparison with NiS_*x*_ in the range of 400–540 cm^−1^.

To elucidate the chemical bonding and functional groups present in the synthesized materials, Fourier-transform infrared (FTIR) spectroscopy was conducted in the wavenumber range of 400–4000 cm^−1^, as shown in Fig. 2. For NiO (Fig. 2(a)), prominent peaks were observed at 408, 426, 551, 1355, and 1637 cm^−1^. The peaks at 408, 426, and 1355 cm^−1^ are attributed to the stretching vibrations of Ni–O bonds, characteristic of the cubic NiO structure. The peak at 551 cm^−1^ and a broad peak around 3500 cm^−1^ correspond to the O–H stretching vibration of adsorbed water molecules on the NiO surface, while the peak at 1637 cm^−1^ is associated with the bending vibration of H–O–H, likely due to moisture exposure during sample preparation in an open environment.^25,42–45^ The presence of these peaks confirms the formation of NiO and highlights the influence of surface-adsorbed species on its vibrational properties. For the NiS_*x*_ sample (Fig. 2(a and c)), FTIR spectra revealed peaks at 400, 610, 1053, and 1631 cm^−1^, with additional peaks in the 400–540 cm^−1^ range at 409, 424, 431, 445, 460, 467, 501, 509, and 534 cm^−1^. The peaks between 400 and 610 cm^−1^ are assigned to the symmetric stretching modes of Ni–S bonds, characteristic of both NiS and NiS_2_. The peak at 1053 cm^−1^ corresponds to the asymmetric stretching mode of Ni–S, indicating the presence of sulfur-based bonding in the nickel sulfides. The small hump at 1631 cm^−1^ is attributed to the O–H vibration of adsorbed water molecules, consistent with the NiO spectra.^30,46–48^ The multiple peaks in the low-wavenumber region suggest a complex bonding environment due to the mixed NiS and NiS_2_. The FTIR spectra of the NiS_*x*_/NiO/MoS_2_ (*x* = 1, 2) heterostructure (Fig. 2(a–c)) exhibit peaks at 416, 426, 437, 457, 478, 524, 683, 863, and 931 cm^−1^. The peaks at 426, 437, 457, 478, 863, and 931 cm^−1^ are attributed to Mo–S stretching and bending vibrations, as well as S–S vibrations in MoS_2_, confirming its presence in the composite. The peak at 524 cm^−1^ corresponds to terminal S–S vibrations, while the peak at 683 cm^−1^ is associated with S–S bonds in metal sulfides. The peak at 416 cm^−1^ is a slightly shifted Ni–O vibration from 408 cm^−1^, indicating interactions between NiO and other components in the composite. The peak at 426 cm^−1^ may also be assigned to NiO, consistent with its presence in the composite.^6,40,49,50^ The coexistence of Ni–O, Ni–S, and Mo–S vibrational modes in the composite spectra confirms the successful integration of NiO, NiS_*x*_, and MoS_2_, with potential interfacial interactions enhancing the chemical properties of material.

### 3.3. Morphological and compositional analysis by SEM and EDX

**Fig. 3:**
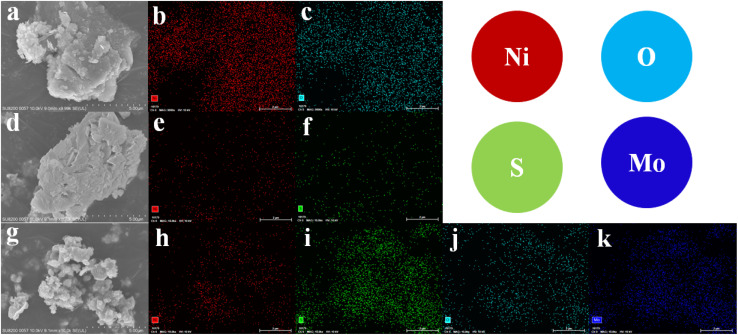
SEM and EDX images of (a–c) NiO, (d–f) NiS_*x*_ (*x* = 1, 2), and (g–k) NiS_*x*_/NiO/MoS_2_ (*x* = 1, 2) heterostructure.

The surface morphology and elemental composition of the synthesized materials were investigated using scanning electron microscopy (SEM) and energy-dispersive X-ray (EDX) spectroscopy, as shown in Fig. 3. The SEM micrograph of NiO (Fig. 3(a)) reveals an agglomerated flake-like structure, indicative of the layered growth typical of hydrothermally synthesized metal oxides. The corresponding EDX spectra (Fig. 3(b and c)) confirm the presence of nickel (Ni) and oxygen (O), with no detectable impurities, verifying the purity of the NiO sample.

For the NiS_*x*_ sample (Fig. 3(d)), the SEM images show agglomerated flake-like structures of varying sizes and shapes, reflecting the mixed-phase nature of NiS and NiS_2_. The EDX spectra (Fig. 3(e and f)) confirm the presence of nickel (Ni) and sulfur (S), consistent with the formation of nickel sulfides. The absence of other elements further validates the purity of the NiS_*x*_ sample. The SEM micrograph of the NiS_*x*_/NiO/MoS_2_ (*x* = 1, 2) heterostructure (Fig. 3(g)) displays an irregular morphology composed of sheet-like structures in random orientations, suggesting the integration of the MoS_2_ layered structure with NiO and NiS_*x*_. This morphology is advantageous for catalytic applications due to the increased surface area and exposure of active edge sites. The EDX spectra (Fig. 3(h–k)) confirm the presence of nickel (Ni), oxygen (O), sulfur (S), and molybdenum (Mo), validating the successful formation of the ternary composite. The random orientation of sheets in the composite may enhance its electrocatalytic performance by providing more accessible active sites.^40^

### 3.4. X-ray photoelectron spectroscopy (XPS) analysis

**Fig. 4:**
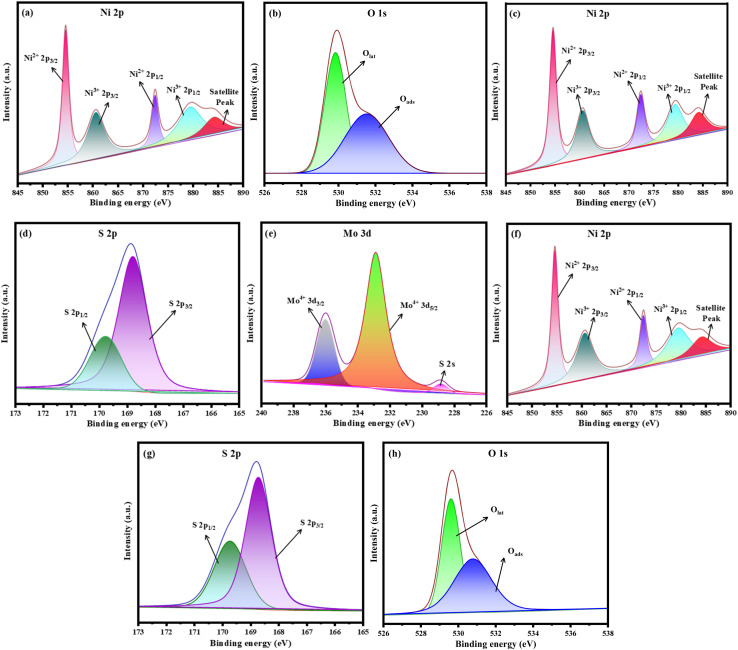
XPS spectra, (a) Ni 2p and (b) O 1s regions of pristine NiO, (c) Ni 2p and (d) S 2p regions of NiS_*x*_ (*x* = 1, 2), (e) Mo 3d, (f) Ni 2p, (g) S 2p, and (h) O 1s regions of the NiS_*x*_/NiO/MoS_2_ (*x* = 1, 2) heterostructure.

The surface chemistry and bonding states of the prepared samples were investigated using high-resolution XPS measurements, as shown in Fig. 4(a–h). All XPS results were fitted using a Shirley-type background subtraction technique, and the background functions for the various element spectroscopies were 80% Gaussian and 20% Lorentz. The peaks depicted in Fig. 4(a and b) confirm the XPS spectra of the NiO nanomaterials. This figure clearly illustrates the presence of Ni and O species, with Ni 2p at around 854.52 eV and 872.44 eV of Ni 2p_3/2_ and 860.56 and 879.37 eV of Ni 2p_1/2_, confirming the presence of Ni^2+^. The high-resolution results of O 1s (Fig. 4(b)) show two peaks at binding energies of 529.83 eV and 531.57 eV, and these peaks are allocated to O_lat_ and O_ads_, respectively.^51,52^ The XPS high-resolution scan Ni 2p and S 2p spectra of NiS_*x*_ (*x* = 1, 2) are presented in Fig. 4(c and d). Compared to the Ni 2p spectrum of NiO, the two doublets Ni 2p_3/2_ and Ni 2p_1/2_ are shifted towards higher binding energies, which are characteristic of pyrite. The high-resolution XPS spectrum of S is presented in Fig. 4(d), and the two doublets, S 2p_3/2_ and S 2p_1/2_, are attributed to the existence of S^2−^ and S_2_^2−^, respectively. Furthermore, Fig. 4(e–h) shows the XPS spectra of the NiS_*x*_/NiO/MoS_2_ (*x* = 1, 2) heterostructure. The figure clearly shows the existence of Ni, S, and O species, and apart from the presence of these species in the ternary composite, Mo 3d and S 2p core-level peak regions existed. The Mo 3d spectrum shows two peaks, with the first two attributed to the doublet Mo 3d_3/2_ and Mo 3d_5/2_, respectively, correlating to the Mo^4+^ state in MoS_2_.^53^ Thus, these results indicate the successful formation of the NiS_*x*_/NiO/MoS_2_ (*x* = 1, 2) heterostructure.

### 3.5. Optical properties and bandgap analysis

**Fig. 5:**
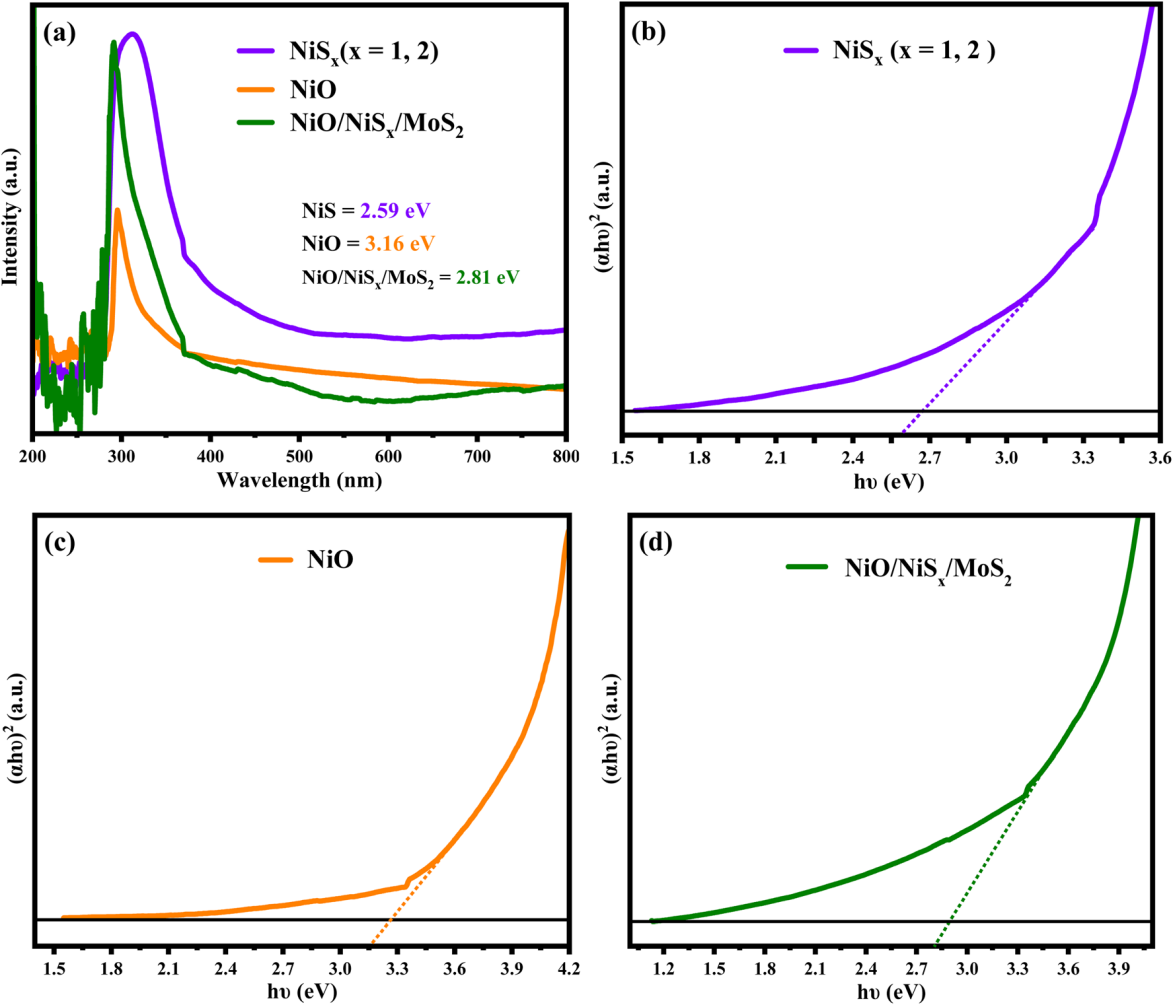
(a) UV-vis absorption spectra and Tauc plots of (b) NiS_*x*_ (*x* = 1, 2), (c) NiO, and (d) NiS_*x*_/NiO/MoS_2_ (*x* = 1, 2) heterostructure.

The optical properties of the synthesized materials were evaluated using UV-visible spectroscopy in the range of 280–500 nm, as shown in Fig. 5(a). The absorption spectra provide insights into the electronic structure and energy transitions of the materials, which are critical for their photocatalytic and electrocatalytic applications. The absorption edge region, corresponding to transitions between the valence and conduction bands, is influenced by structural disorder, defects, and lattice distortions, which affect the precise determination of the bandgap energy (*E*_g_).^54^ The bandgap energy was calculated using the Tauc equation:

1 (*αhν*)^1/2^ = *A*(*hν* − *E*_g_)

where *α* is the optical absorption coefficient (2.303), *ν* is the photon energy, *A* is a constant, and *E*_g_ is the bandgap energy. The Tauc plots for NiO, NiS_*x*_, and NiS_*x*_/NiO/MoS_2_ (*x* = 1, 2) heterostructure are shown in Fig. 5(b–d), yielding bandgap energies of 3.16 eV, 2.59 eV, and 2.81 eV, respectively. The bandgap of NiO is consistent with its wide-bandgap semiconductor nature, suitable for applications in alkaline environments.^54^ The lower bandgap of NiS_*x*_ reflects the mixed-phase composition of NiS and NiS_2_, which introduces additional electronic states that reduce the energy gap. The NiS_*x*_/NiO/MoS_2_ (*x* = 1, 2) heterostructure exhibits an intermediate bandgap of 2.81 eV, lying between those of NiO and NiS_*x*_. This reduction in bandgap is attributed to synergistic interactions among the components, which modify the electronic structure by introducing intermediate states near the conduction and valence bands. These states facilitate charge carrier transfer, enhancing the suitability of the material for electrocatalytic applications such as the hydrogen evolution reaction (HER).^55^

The formation of heterojunctions in the composite aligns the energy bands of NiO, NiS_*x*_, and MoS_2_, creating pathways for efficient charge transfer. The increased surface area and exposure of active edge sites in the composite further enhance its catalytic efficiency by facilitating proton adsorption and charge transfer, making it a promising candidate for photocatalysis and HER applications.^54^

### 3.6. Electrochemical performance analysis

#### 3.6.1. Cyclic voltammetry

**Fig. 6:**
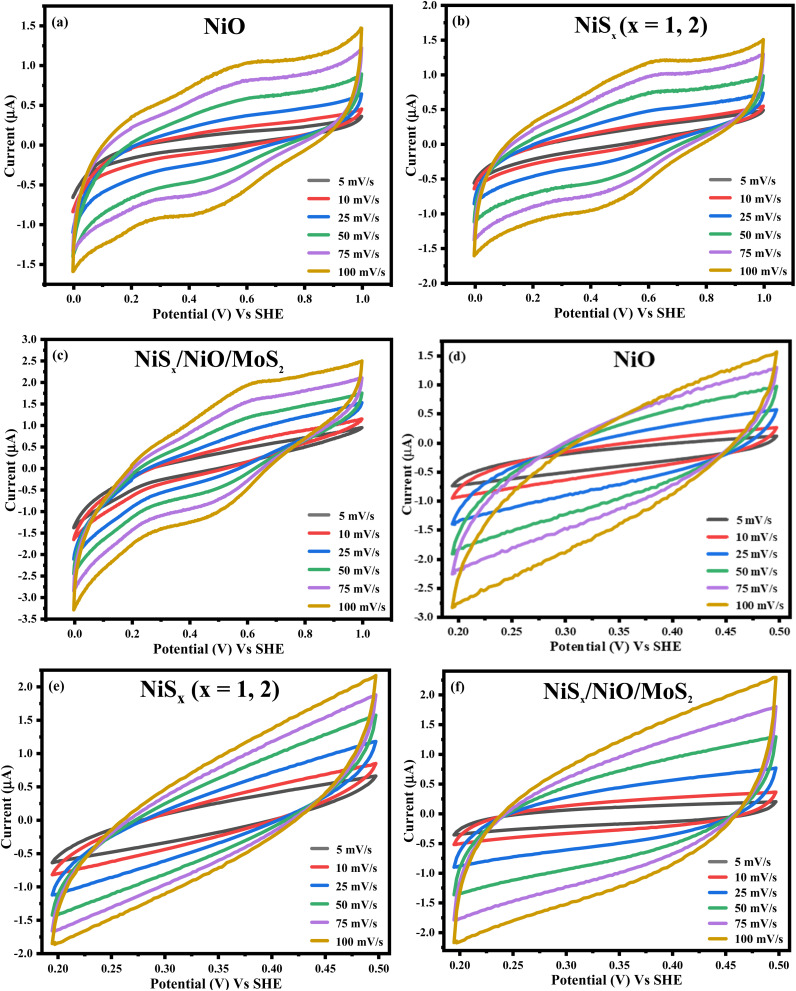
CV of (a) NiO, (b) NiS_*x*_, and (c) NiS_*x*_/NiO/MoS_2_ (*x* = 1, 2) heterostructure at different scan rates. CV of (d) NiO, (e) NiS_*x*_ (*x* = 1, 2), and (f) NiS_*x*_/NiO/MoS_2_ (*x* = 1, 2) heterostructure at a smaller potential window.

The electrochemical properties of NiO, NiS_*x*_, and NiS_*x*_/NiO/MoS_2_ (*x* = 1, 2) heterostructure were investigated using a three-electrode setup in a 1 M H_2_SO_4_ electrolyte. Cyclic voltammetry (CV) curves were recorded over a potential window of 0 to 1 V *vs.* Standard Hydrogen Electrode (SHE) at various scan rates, as shown in Fig. 6(a–c) and Table S1. The CV curves exhibit distinct redox peaks, indicating a pseudocapacitive (faradaic) behaviour attributed to the reversible redox transitions between Ni^2+^ and Ni^3+^. These transitions enable fast faradaic reactions, contributing to charge storage and electrocatalytic activity.^41,55–57^ The presence of redox peaks confirms the ability of materials to undergo reversible redox reactions, making them suitable for energy storage and electrocatalysis.

At higher scan rates, the current density increases, reflecting a higher diffusion rate compared to the reaction rate, which is typical for pseudocapacitive materials.^40,58,59^ The NiS_*x*_/NiO/MoS_2_ (*x* = 1, 2) heterostructure shows enhanced redox peak intensity compared to NiO and NiS_*x*_, suggesting improved charge storage capacity due to the incorporation of MoS_2_. The layered structure of MoS_2_ increases the number of active sites and enhances electrical conductivity, facilitating faster charge transfer.^60^

To determine the electrochemically active surface area (ECSA), the double-layer capacitance (*C*_dl_) was calculated from CV measurements in the non-faradaic region (1.969 to 0.5 V *vs.* SHE) at varying scan rates in a 1 M KOH electrolyte, as shown in Fig. 6(d–f). The charging current density was plotted against scan rates, and the slope of the resulting graph (Fig. 8(a)) was used to calculate *C*_dl_. The ECSA was determined using the equation:

2 ECSA = *C*_dl_/*C*_s_

where *C*_s_ is the specific capacitance of the material. The calculated ECSA values for NiO, NiS_*x*_, and NiS_*x*_/NiO/MoS_2_ (*x* = 1, 2) heterostructure were 0.3, 0.5, and 0.7 mF cm^−2^, respectively. The heterostructure exhibited the highest ECSA, indicating a larger surface area available for electrochemical reactions. This enhancement is attributed to the high surface area of MoS_2_ and NiS_2_, which expose more active edge sites, facilitating proton adsorption and charge transfer.^60,61^

**Fig. 7:**
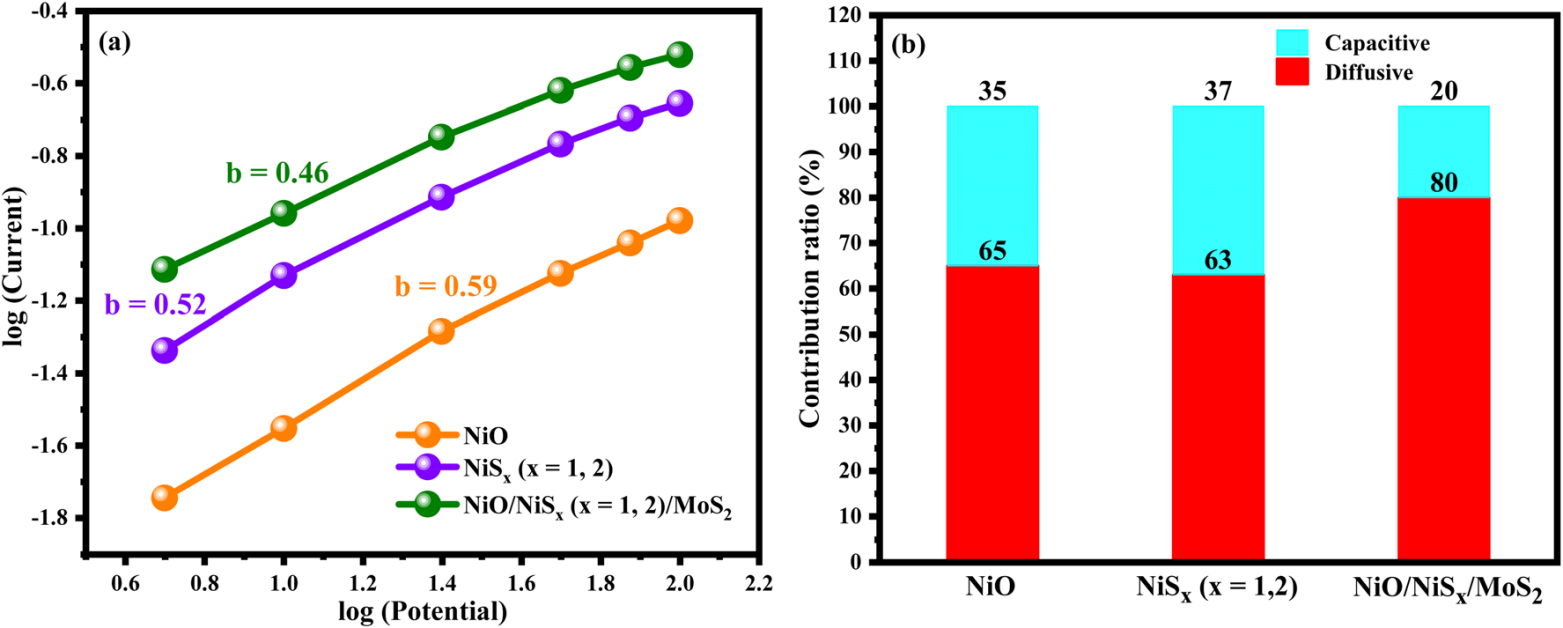
log(*i*) *vs.* log(*v*) curves of (a) NiO, NiS_*x*_ (*x* = 1, 2), and NiS_*x*_/NiO/MoS_2_ (*x* = 1, 2) heterostructure, and (b) comparison of capacitive and diffusive controlled regions.

For further investigation, the capacitive and diffusion-controlled contributions were analyzed using the Dunn method to examine the capacitive and diffusion-controlled contributions to the charge storage mechanism of the prepared electrodes. The current response was gathered at a fixed potential of 0.5 V and was fitted to the equation:

3 *i*(*V*) = *k*_1_*v* + *k*_2_*v*^1/2^

where the first term is the capacitive current and the second term is the current due to diffusion-controlled processes. Fig. 7, shows that NiO exhibited a mixed capacitive and diffusion-controlled mechanism with approximately 35% capacitive and 65% diffusion contribution. NiS_*x*_ showed a similar mixed mechanism with capacitive contributions of about 37%. However, the NiS_*x*_/NiO/MoS_2_ (*x* = 1, 2) heterostructure showed a dominating diffusion-controlled mechanism, reaching a diffusion contribution of 80 to 100% across all scan rates. To further support these observations, the *b*-value was determined from the power law relationship.

4 *i* = *av*^*b*^

where *i* is the current, *v* is the voltage used in CV data, *a* and *b* are constants, and the slope of the log(*i*) *vs.* log(*v*) plot yields the *b*-value. A *b*-value close to 1 indicates a surface-controlled capacitive process, whereas a value near 0.5 indicates a diffusion-controlled mechanism. The *b*-values, derived from the slope of log(*i*) *versus* log(*v*) plots, also support these findings: *b* = 0.59 for NiO, *b* = 0.52 for NiS_*x*_, and *b* = 0.46 for the NiS_*x*_/NiO/MoS_2_ (*x* = 1, 2) heterostructure. The closer *b*-values that are near 0.5 show more diffusion-controlled processes, supporting the observation from Dunn's method suggests that the storage mechanism of the composite electrode is primarily redox-driven. The greater the diffusion contribution is, the more it is attributed to the composite's enhanced synergistic effect of ionic and electronic coupling.^62,63^

The calculated *b*-values and average capacitive and diffusive controlled contributions percentages with their interpretations are given in Table 1.

**Table 1:** Details of % capacitive and % diffusive controlled contributions

Sample names	*b*-Value	% capacitive (average)	% diffusive (average)	Interpretation
NiO	0.59	35	65	Mix, slightly capacitive leaning
NiS_*x*_ (*x* = 1, 2)	0.52	37	63	Mix, more diffusive
NiS_*x*_/NiO/MoS_2_	0.46	20	80	Strongly diffusive-controlled

#### 3.6.2. Electrochemical impedance spectroscopy (EIS)

**Fig. 8:**
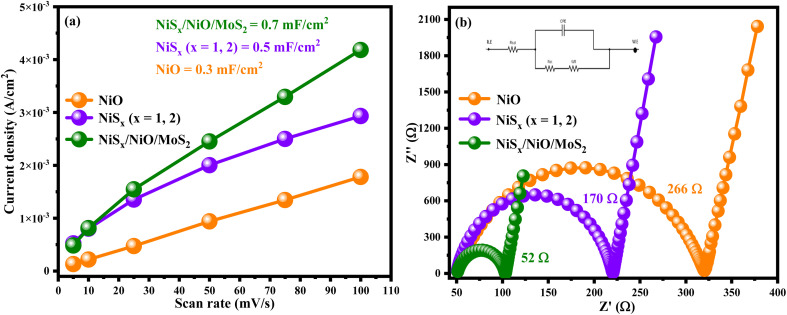
(a) ECSA and (b) EIS Nyquist plots along with the equivalent circuit of NiO, NiS_*x*_ (*x* = 1, 2), and NiS_*x*_/NiO/MoS_2_ (*x* = 1, 2) heterostructure.

Electrochemical impedance spectroscopy (EIS) was performed in the frequency range of 10^6^ to 10^−6^ Hz under open-circuit conditions to assess the electrical conductivity and charge transfer properties of the samples. The Nyquist plots and the equivalent circuit used for fitting are shown in Fig. 8(b). The equivalent circuit consists of a bulk solution resistance (*R*_s_ = 2–5 Ω), a charge-transfer resistance (*R*_ct_), a Warburg diffusion element (*W*_R_), and a constant phase element (CPE) representing the double-layer capacitance. The EIS data were fitted using ZView 4.0 software to determine *R*_ct_ values, which were 266, 170, and 52 Ω for NiO, NiS_*x*_ (*x* = 1, 2), and NiS_*x*_/NiO/MoS_2_ (*x* = 1, 2) heterostructure, respectively. The significantly lower *R*_ct_ value of the heterostructure indicates enhanced charge transfer kinetics, likely due to the synergistic effects of MoS_2_ high conductivity and the increased active sites provided by the composite heterostructure. The reduced *R*_ct_ facilitates faster electron transfer, which is critical for efficient electrocatalytic performance in HER.^64^

#### 3.6.3. Galvanostatic charge–discharge (GCD) analysis

**Fig. 9:**
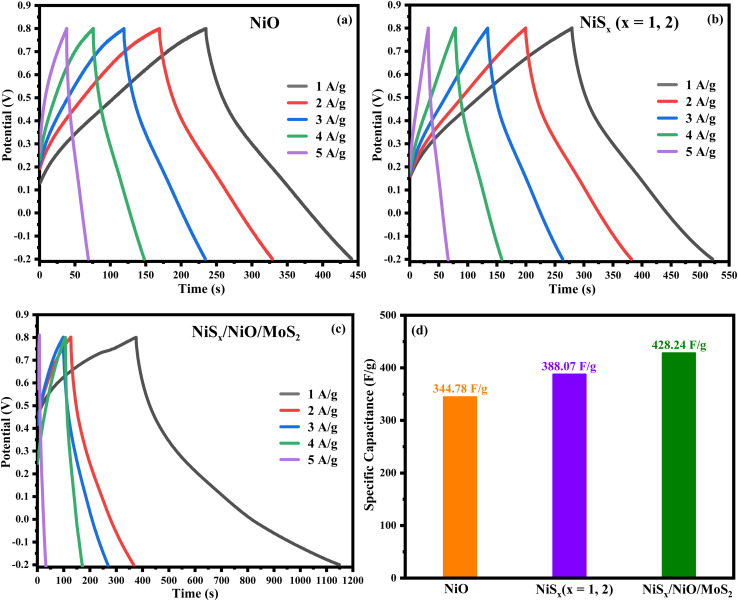
GCD curves of (a) NiO, (b) NiS_*x*_ (*x* = 1, 2), and (c) NiS_*x*_/NiO/MoS_2_ (*x* = 1, 2) heterostructure at various current densities, and (d) comparison of calculated specific capacitances from GCD curves.

Galvanostatic charge–discharge (GCD) curves were recorded in the potential range of 0–1 V at current densities ranging from 1 to 5 A g^−1^, as shown in Fig. 9(a–c). The GCD curves exhibit a nonlinear, triangular shape, indicative of pseudocapacitive behaviour due to faradaic reactions involving Ni^2+^ and Ni^3+^ redox pairs. The specific capacitance (*C*_s_), energy density (*E*), and power density (*P*) were calculated using the following equations:

5 *C*_s_ = (*I* × Δ*t*)/(*m* × Δ*V*)

6 *E* = (*C*_s_ × Δ*V*^2^ × 0.5)/3.6

7 *P* = *E* × 3600/Δ*t*

where *I* is the applied current, Δ*t* is the discharge time, *m* is the active mass of the sample, and Δ*V* is the potential window. The units of *C*_s_, *E*, and *P* in eqn (5), (6), and (7) is F g^−1^, Wh kg^−1^, and W kg^−1^, respectively. The calculated values are summarized in Table 2. The NiS_*x*_/NiO/MoS_2_ (*x* = 1, 2) heterostructure exhibited the highest specific capacitance of 428.24 F g^−1^, energy density of 356.87 Wh kg^−1^, and power density of 9000 W kg^−1^ at 2 A g^−1^, as shown in Fig. 9(d). These values are significantly higher than those of NiO (344.78 F g^−1^, 287.32 Wh kg^−1^) and NiS_*x*_ (388.07 F g^−1^, 323.39 Wh kg^−1^), showing the superior energy storage capacity of the composite. The enhanced performance of the NiS_*x*_/NiO/MoS_2_ (*x* = 1, 2) heterostructure is attributed to the pseudo-capacitance generated by the Ni^2+^ and Ni^3+^ redox reactions, combined with the high surface area of MoS_2_ and NiS_*x*_. The composite structure facilitates the formation of a Helmholtz double layer, enhancing charge storage and transfer efficiency.^64^

**Table 2:** Comparison of the specific capacitance, energy density, and power density of NiO, NiS_*x*_ (*x* = 1, 2), and NiS_*x*_/NiO/MoS_2_ (*x* = 1, 2) heterostructure

Sample names	Specific capacitance (F g^−1^)	Energy density (Wh kg^−1^)	Power density (W kg^−1^)
NiO	344.7825	287.3187	7000
NiS_*x*_ (*x* = 1, 2)	388.0728	323.394	8000
NiS_*x*_/NiO/MoS_2_	428.2425	356.8687	9000

#### 3.6.4. Linear sweep voltammetry (LSV) and Tafel slope analysis

**Fig. 10:**
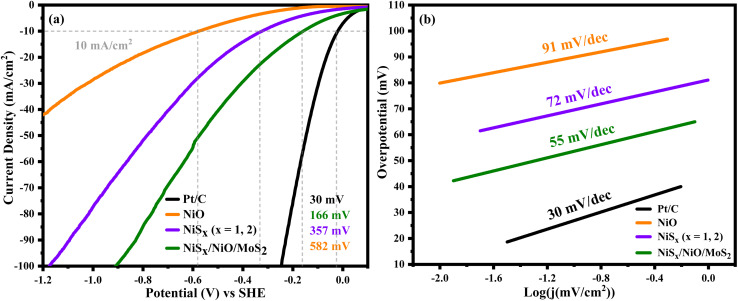
Comparison of the electrochemical HER performances (a) polarization curves at a scan rate of 5 mV s^−1^, and (b) Tafel plots of NiO, NiS_*x*_ (*x* = 1, 2), and NiS_*x*_/NiO/MoS_2_ (*x* = 1, 2) heterostructure in 1 M H_2_SO_4_.

**Fig. 11:**
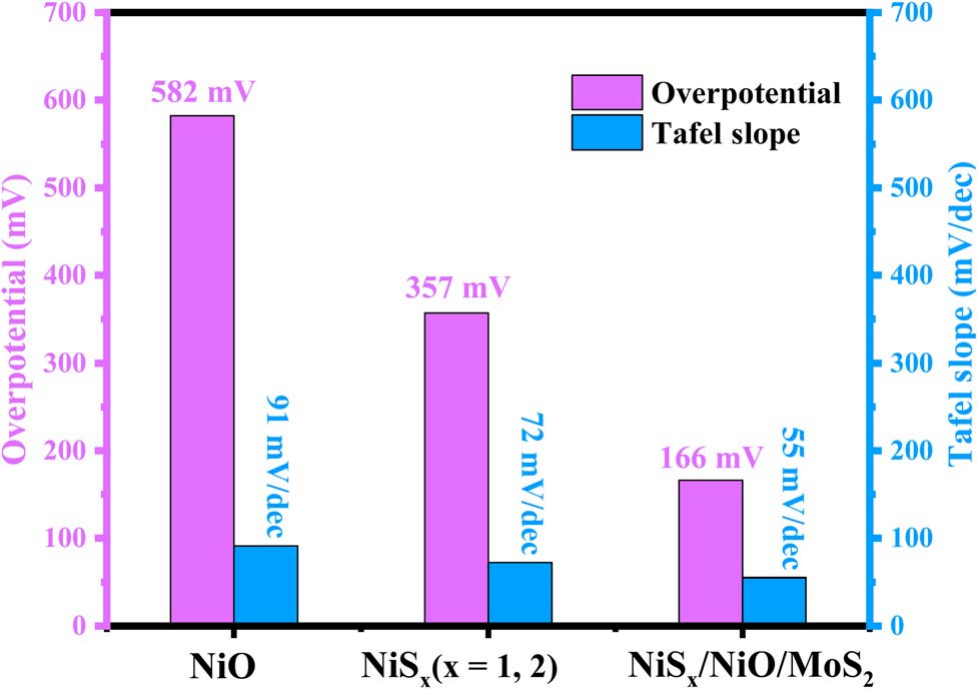
Comparison of overpotential and Tafel slope of NiO, NiS_*x*_, and NiS_*x*_/NiO/MoS_2_ (*x* = 1, 2) heterostructure.

The electrocatalytic performance for the hydrogen evolution reaction (HER) was evaluated using *iR*-corrected linear sweep voltammetry (LSV) in a 1 M H_2_SO_4_ electrolyte at a scan rate of 5 mV s^−1^, with a platinum electrode as a reference, as shown in Fig. 10(a). The LSV curves indicate that NiO and NiS_*x*_ achieved a mass-normalized current density of 10 mA cm^−2^ at an overpotential of 582 mV and 357 mV, respectively, while the NiS_*x*_/NiO/MoS_2_ (*x* = 1, 2) heterostructure required a significantly lower overpotential of 166 mV. This superior performance of the composite is attributed to its synergistic composition, which enhances charge transfer and increases the number of active sites, particularly the edge sites of MoS_2_ and NiS_2_.^40^ To further assess the HER kinetics, Tafel slope analysis was performed using the Tafel equation:

8 *η* = *β* log *j* + *a*

where *η* is the overpotential, *j* is the exchange current density, *β* is the Tafel slope, and a is a constant. The Tafel slopes for NiO, NiS_*x*_, and NiS_*x*_/NiO/MoS_2_ (*x* = 1, 2) heterostructure were 91, 72, and 55 mV dec^−1^, respectively, as shown in Fig. 10(b), and comparison of overpotential and Tafel slope in Fig. 11, and summarized in Table 3. The HER in acidic media involves three primary steps:H^+^ + e^−^ → H_ads_ [Volmer step (120 mV dec^−1^)]H^+^ + H_ads_ + e^−^ → H_2_ [Heyrovsky step (40 mV dec^−1^)]H_ads_ → H_2_ [Tafel step (30 mV dec^−1^)]

**Table 3:** Comparison of overpotential, Tafel slope at mass-normalized current density of 10 mA cm^−2^, and charge transfer resistance 1 M H_2_SO_4_

Sample names	Overpotential (mV)	Tafel slope (mV dec^−1^)	Charge transfer resistance (*R*_ct_) (Ω)
NiO	582	91	266
NiS_*x*_ (*x* = 1, 2)	357	72	170
NiS_*x*_/NiO/MoS_2_	166	55	52

The Tafel slope of 55 mV dec^−1^ for NiS_*x*_/NiO/MoS_2_ (*x* = 1, 2) heterostructure suggests that the HER follows a Volmer–Heyrovsky mechanism, with the Heyrovsky step (desorption of H_2_) as the rate-limiting step. The low Tafel slope indicates favourable electron transfer kinetics, making the composite a highly effective HER catalyst.^65,66^ Further, the durability of the as-prepared composite electrode was evaluated through multi-cycle LSV stability tests. Approximately 700 consecutive LSV cycles were performed, during which only a negligible shift in overpotential was observed, indicating excellent electrochemical stability. To further substantiate this observation, we have included LSV curves of the composite recorded before and after 700 cycles under identical experimental conditions in the SI (Fig. S6). The nearly overlapping polarization curves clearly demonstrate minimal degradation in overpotential and current density, confirming the structural integrity and robust electrocatalytic behaviour of the composite in an acidic environment. These consistent LSV responses indicate that the composite maintains stable HER activity during repeated electrochemical operation, highlighting its suitability for practical HER applications.

**Fig. 12:**
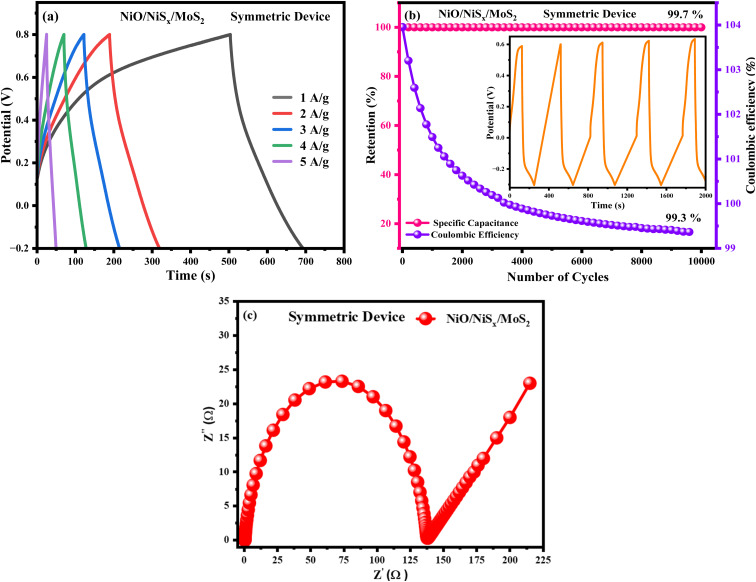
(a) GCD curves, (b) retention and coulombic efficiency, and (c) EIS curves of the NiS_*x*_/NiO/MoS_2_ symmetric device.

The cyclic stability performance and coulombic efficiency of the fabricated symmetric device were evaluated using galvanostatic charge–discharge (GCD) analysis. As shown in Fig. 11(a and b), the GCD curves obtained at current densities of 1–5 A g^−1^ revealed nearly symmetric triangular shapes, confirming excellent charge–discharge reversibility and efficient electrochemical kinetics. At a current density of 1 A g^−1^, the device achieved a high volumetric capacitance of 250.2 F g^−1^, calculated from the discharge time. The specific capacitance (*C*_s_ = 250 F g^−1^), energy density (*E* = 55 Wh kg^−1^), and power density (*P* = 495 W kg^−1^) were calculated using eqn (5), (6), and (7), respectively. These computed values demonstrate the excellent electrochemical performance of the composite-based electrode. The specific capacitance retention measurements were performed to evaluate the cyclic stability and coulombic efficiency of the fabricated devices at 10 000 GCD cycles. As shown in Fig. 12(b), the symmetric device (Fig. S7) has high specific cyclic stability retentions of 99.7%, in contrast with coulombic efficiencies of 99.3%, respectively, confirming its outstanding durability and excellent charge transfer efficiency over prolonged cycling. Such high stability is indicative of the robust structural reliability and reversibility of the redox processes within the electrode material. Moreover, electrochemical impedance spectroscopy (EIS) was performed after the cycling test to gain insights into the interfacial characteristics of the device. The Nyquist plot shown in Fig. 12(c) shows a small charge transfer resistance (*R*_ct_) of 88.7 Ω, which can be attributed to the highly conductive and interconnected network of the NiS_*x*_/NiO/MoS_2_ heterostructure and the well-optimized electrode–electrolyte interface.

Additionally, the near-vertical Warburg tail in the low-frequency region suggests efficient ion diffusion and confirms the presence of hierarchical porosity in the electrode material. This structural feature plays a key role in facilitating rapid ion transport and enhancing the overall electrochemical performance of the device.

### 3.7. Discussion of electrochemical performance

The superior electrochemical performance of the NiS_*x*_/NiO/MoS_2_ (*x* = 1, 2) heterostructure is attributed to several factors. First, the high ECSA (0.7 mF cm^−2^) reflects an increased number of active sites, particularly the edge sites of MoS_2_ and NiS_2_, which are known to be catalytically active for HER.^60^ The low *R*_ct_ (52 Ω) indicates enhanced charge transfer kinetics, facilitated by the high conductivity of NiS_*x*_ and the layered structure of MoS_2_. The redox activity of the Ni^2+^ and Ni^3+^ pair contributes to pseudocapacitive behaviour, enhancing charge storage and catalytic efficiency.^41,61^

The incorporation of MoS_2_ in the composite introduces additional active sites and improves electrical conductivity, as evidenced by the reduced bandgap (2.81 eV) and lower Tafel slope (55 mV dec^−1^). The heterojunctions formed between NiO, NiS_*x*_, and MoS_2_ create intermediate electronic states that facilitate charge carrier mobility, reducing energy barriers for electron transfer. The Mo^4+^ ions in MoS_2_ act as trap centres, further enhancing charge transfer and catalytic activity.^64,67^ Compared to NiO and NiS_*x*_, the enhanced performance of the composite is evident in its lower overpotential (166 mV *vs.* 582 mV for NiO and 357 mV for NiS_*x*_), indicating a lower energy requirement for HER. The Tafel slope of 55 mV dec^−1^ suggests faster reaction kinetics, approaching the performance of platinum-based catalysts, which typically exhibit Tafel slopes of 30–40 mV dec^−1^.^65^ The high specific capacitance (428.24 F g^−1^) and energy density (356.87 Wh kg^−1^) further highlight the potential of the composite for energy storage applications, making it a versatile material for both electrocatalysis and supercapacitors.

## 4. Conclusion

The NiS_*x*_/NiO/MoS_2_ (*x* = 1, 2) heterostructure was successfully synthesized through a hydrothermal method and demonstrated exceptional performance as an electrocatalyst for the hydrogen HER in 1 M H_2_SO_4_ and as an electrode material for supercapacitor applications. The composite exhibits a low overpotential of 166 mV at a mass-normalized current density of 10 mA cm^−2^, a Tafel slope of 55 mV dec^−1^, and a charge transfer resistance of 52 Ω for HER, significantly outperforming individual NiO (582 mV, 91 mV dec^−1^, 266 Ω) and NiS_*x*_ (357 mV, 72 mV dec^−1^, 170 Ω). Additionally, it achieves a high specific capacitance of 428.24 F g^−1^ at 2 A g^−1^ and an ECSA of 0.7 mF cm^−2^, compared to 344.78 F g^−1^ for NiO and 388.07 F g^−1^ for NiS_*x*_. These enhancements are attributed to synergistic interactions among NiS_*x*_, NiO, and MoS_2_, which increase active site density, improve charge transfer efficiency, and enhance electrochemical stability. The Tafel slope of 55 mV dec^−1^ is competitive with Pt-based catalysts (30–40 mV dec^−1^) and comparable to other non-noble metal catalysts such as NiS_2_/MoS_2_ (60 mV dec^−1^) and NiCoP (55 mV dec^−1^), underscoring its potential as a cost-effective alternative for hydrogen production. Furthermore, the high specific capacitance and large ECSA highlight the viability of heterostructures for energy storage applications. The specific capacitance retention measurements were performed to evaluate the cyclic stability and coulombic efficiency of the fabricated device at 10 000 GCD cycles. The symmetric device has high specific cyclic stability retentions of 99.7%, in contrast with coulombic efficiency of 99.3%, respectively, confirming its outstanding durability and excellent charge transfer efficiency over prolonged cycling. Such high stability is indicative of the robust structural reliability and reversibility of the redox processes within the electrode material. This study positions the NiS_*x*_/NiO/MoS_2_ (*x* = 1, 2) heterostructure as a promising dual-functional material for scalable hydrogen evolution and supercapacitor technologies, contributing to the advancement of sustainable energy systems.

## Ethical statement

The authors declare that the research work presented in this manuscript was conducted in accordance with ethical standards. This research did not involve any studies with human participants or animals performed by any of the authors.

## Author contributions

Sumaiya Saleem: investigation, methodology, writing – original draft. Muhammad Salman: investigation, writing – original draft, software analysis. Ilham Noor: software analysis. Baseena Sardar: data curation, visualization. Majid Khan: conceptualization, supervision, formal analysis, validation, funding acquisition, writing – review and editing.

## Conflicts of interest

The authors declare that they have no known competing financial interests or personal relationships that could influence the work reported in this study.

## Supplementary Material

RA-016-D5RA06931A-s001

## Data Availability

Additional raw/processed data are available from the corresponding author upon reasonable request. Supplementary information (SI): characterization data (EDX, SEM), electrochemical performance results (CV, GCD, LSV), synthesis schematics, comparative performance tables, and device schematics for the NiS_*x*_/NiO/MoS_2_ heterostructure. See DOI: https://doi.org/10.1039/d5ra06931a.

## References

[cit1] Pal B., Yang S., Ramesh S., Thangadurai V., Jose R. (2019). Nanoscale Adv..

[cit2] Winter C. J. (2009). Int. J. Hydrogen Energy.

[cit3] Faber M. S., Lukowski M. A., Ding Q., Kaiser N. S., Jin S. (2014). J. Phys. Chem. C.

[cit4] Ye J., Zang Y., Wang Q., Zhang Y., Sun D. (2021). et al.. J. Energy Chem..

[cit5] Kibsgaard J., Chen Z., Reinecke B. N., Jaramillo T. F. (2012). Nat. Mater..

[cit6] Saleem S., Salman M., Ali S., Ling Y., Khan M. (2022). Int. J. Hydrogen Energy.

[cit7] Zhang N., Wang B., Hu P., Gao Z., Wang H. (2025). J. Environ. Chem. Eng..

[cit8] Yamakov V. I., Rains A. A., Kang J. H., Das L., Rashid R. (2023). et al.. ACS Appl. Mater. Interfaces.

[cit9] Zhu L., Song Y., Chen H., Wang M., Liu Z. (2025). et al.. Int. J. Hydrogen Energy.

[cit10] Zhang Y., Ouyang B., Xu K., Xia X., Zhang Z. (2018). et al.. Small.

[cit11] Chandrasekaran S., Yao L., Deng L., Bowen C., Zhang Y. (2019). et al.. Chem. Soc. Rev..

[cit12] Liu J., Liu Y., Liu N., Han Y., Zhang X. (2015). et al.. Science.

[cit13] Hisatomi T., Kubota J., Domen K. (2014). Chem. Soc. Rev..

[cit14] Kudo A., Miseki Y. (2009). Chem. Soc. Rev..

[cit15] Ghasemi F., Jalali M., Abdollahi A., Mohammadi S., Sanaee Z. (2017). et al.. RSC Adv..

[cit16] Dai Y., Wen X., Yu W., Huang X., Liu J. (2025). et al.. Chem.–Asian J..

[cit17] Ren X. Q., Chen M. X., Cao X., Dai Y. L., Yu W. X. (2025). et al.. Rare Met..

[cit18] Liu Z., Jin R., Zhao G., Tang Q., Kang T. (2025). et al.. Mater. Horiz..

[cit19] Ghafoor S., Noor Y., Salman M., Saleem S., Ullah A. (2025). et al.. ChemistrySelect.

[cit20] Zhou J. E., Chen J., Peng Y., Zheng Y., Zeb A., Lin X. (2022). Coord. Chem. Rev..

[cit21] Tian Y., Yang X., Nautiyal A., Zheng Y., Guo Q. (2019). et al.. Adv. Compos. Hybrid Mater..

[cit22] Ray S. C. (2000). J. Mater. Sci. Lett..

[cit23] Liu L., Li X., Xu L. C., Liu R., Yang Z. (2017). Appl. Surf. Sci..

[cit24] Wen Y. Y. , Zeng X. B., Chen X. X., Wang W. Z., Ding J. and Xu S. E., Adv. Mater. Eng. Conf. Proc., 2016, pp. 1034–1039

[cit25] Yin Z., Liu X., Chen S., Xie H., Gao L. (2022). et al.. Mater. Today Nano.

[cit26] Shi W., Wang Z. (2018). J. Taiwan Inst. Chem. Eng..

[cit27] He Y., Tang P., Hu Z., He Q., Zhu C. (2020). et al.. Nat. Commun..

[cit28] Seo B., Jung G. Y., Sa Y. J., Jeong H. Y., Cheon J. Y. (2015). et al.. ACS Nano.

[cit29] Pan L., Zhang H., Li L., Yan Z., Ju D. (2017). et al.. ACS Appl. Mater. Interfaces.

[cit30] Jiang N., Tang Q., Sheng M., You B., Jiang D. E., Sun Y. (2016). Catal. Sci. Technol..

[cit31] Peng L., Liang Y., Wu S., Li Z., Sun H. (2022). et al.. J. Alloys Compd..

[cit32] Gong M., Zhou W., Tsai M.-C., Zhou J., Guan M. (2014). et al.. Nat. Commun..

[cit33] Gong M., Wang D. Y., Chen C. C., Hwang B. J., Dai H. (2016). Nano Res..

[cit34] Zhao W., Bajdich M., Carey S., Vojvodic A., Nørskov J. K., Campbell C. T. (2016). ACS Catal..

[cit35] Wu X., Yang B., Li Z., Lei L., Zhang X. (2015). RSC Adv..

[cit36] Kullerud G., Yund R. A. (1962). J. Petrol..

[cit37] Mondal D., Villemure G., Li G., Song C., Zhang J. (2013). et al.. Appl. Catal., A.

[cit38] Arunprasad M., Theerthagiri J., Madhavan J., Murugan K. (2017). Phys. Chem. Chem. Phys..

[cit39] Nikam R. D., Lu A. Y., Sonawane P. A., Kumar U. R., Yadav K. (2015). et al.. ACS Appl. Mater. Interfaces.

[cit40] Amin N. U., Saleem S., Shah A., Salman M., Ling Y., Khan M. (2023). Mater. Sci. Semicond. Process..

[cit41] Su L., Xiao Y., Han G., Lin J.-Y. (2018). J. Nanopart. Res..

[cit42] Qiao H., Wei Z., Yang H., Zhu L., Yan X. (2009). J. Nanomater..

[cit43] Khalaji A. D., Das D. (2014). Int. Nano Lett..

[cit44] Ghaffari A., Behzad M., Pooyan M., Rudbari H. A., Bruno G. (2014). J. Mol. Struct..

[cit45] Haider A. J., Al-Anbari R., Sami H. M., Haider M. J. (2019). Energy Procedia.

[cit46] Fazli Y., Pourmortazavi S. M., Kohsari I., Sadeghpur M. (2014). Mater. Sci. Semicond. Process..

[cit47] Kristl M., Dojer B., Gyergyek S., Kristl J. (2017). Heliyon.

[cit48] Surendran S., Sankar K. V., Berchmans L. J., Selvan R. K. (2015). Mater. Sci. Semicond. Process..

[cit49] Darezereshki E., Vakylabad A. B., Hassanzadeh A., Niedoba T., Surowiak A. (2021). et al.. Minerals.

[cit50] Alamro T., Ram M. K. (2017). Electrochim. Acta.

[cit51] Lai B., Singh S. C., Bindra J. K., Saraj C. S., Shukla A. (2019). et al.. Mater. Today Chem..

[cit52] Salman M., Saleem S., Ling Y., Khan M., Gao Y. (2024). Ceram. Int..

[cit53] Koroteev V. O., Bulusheva L. G., Asanov I. P., Shlyakhova E. V., Vyalikh D. V., Okotrub A. V. (2011). J. Phys. Chem. C.

[cit54] Saleem S., Salman M., Elgorban A. M., Al-Shwaiman H. A., Ling Y., Khan M. (2025). Mater. Sci. Semicond. Process..

[cit55] Salman M., Khan M., Saleem S., Ali S., Hussain F. (2021). et al.. Mater. Today Commun..

[cit56] Huang J. J., Hwang W. S., Weng Y. C., Chou T. C. (2010). Mater. Trans..

[cit57] Meher S. K., Justin P., Rao G. R. (2011). Nanoscale.

[cit58] Wang H., Wang J., Liang M., He Z., Li K. (2021). et al.. ACS Omega.

[cit59] Luo S., Yang M., Li J., Wu Y. (2023). RSC Adv..

[cit60] Shah A., Saleem S., Amin N. U., Salman M., Ling Y. (2023). et al.. Mater. Sci. Eng., B.

[cit61] Liu M., Wang J., Klysubun W., Wang G., Sattayaporn S. (2021). et al.. Nat. Commun..

[cit62] Sharma S., Chand P. (2023). Results Chem..

[cit63] Forghani M., Donne S. W. (2018). J. Electrochem. Soc..

[cit64] Pang H., Wei C., Li X., Li G., Ma Y., Li S. (2014). et al.. Sci. Rep..

[cit65] Wang J. X., Uribe F. A., Springer T. E., Zhang J., Adzic R. R. (2009). Faraday Discuss..

[cit66] Kahyarian A., Brown B., Nesic S. (2017). J. Electrochem. Soc..

[cit67] Chen P., Xu K., Fang Z., Tong Y., Wu J., Lu X. (2015). et al.. Angew. Chem..

