# Supplementary material for: NiSx/NiO/MoS2 heterostructure for enhanced hydrogen evolution in acidic media and supercapacitor electrode applications
Source: RSC Adv. 2026 Jan 14;16(4):3261–75. doi: 10.1039/d5ra06931a (PMC12802422; doi:10.1039/d5ra06931a)
Supplement: RA-016-D5RA06931A-s001 [file RA-016-D5RA06931A-s001.pdf]

Supplementary Information (SI) for RSC Advances.
This journal is © The Royal Society of Chemistry 2026

Supporting Information

$NiS_x/NiO/MoS_2$ Heterostructure for Enhanced Hydrogen Evolution in Acidic Media and Supercapacitor Electrode Applications

Sumaiya Saleem[1, #], Muhammad Salman[1, #], Ilham Noor[2], Baseena Sardar[1], Majid Khan[1, *]

[1] Department of Physics, Abdul Wali Khan University Mardan, Mardan 23200, Pakistan

[2] Department of Physics, University of Malakand, Chakdara, 18800, Pakistan

[#] These two authors equally contributed to this work

[*] Corresponding author: Dr. Majid Khan, majidkhan@awkum.edu.pk

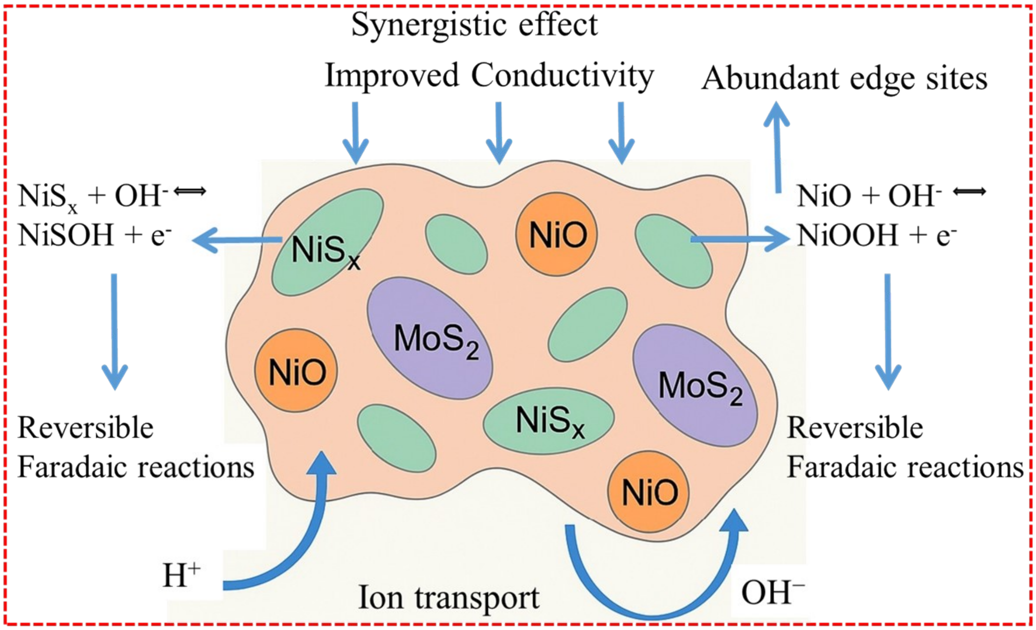

Figure S1: Mechanism of the selected electrode materials based on their redox-active components.

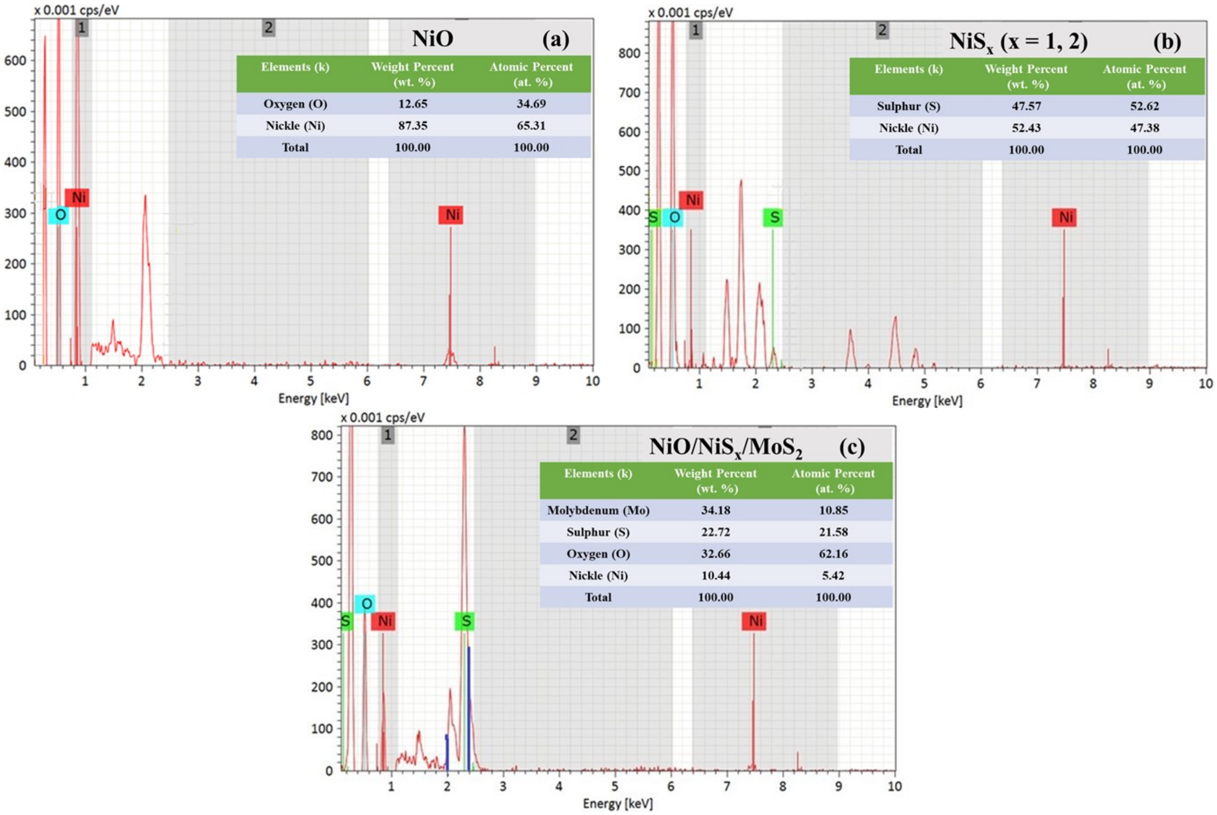

Figure S2: EDX results of (a) NiO, (b) NiS_x, and (c) $NiS_x/NiO/MoS_2$ (x = 1, 2) heterostructure.

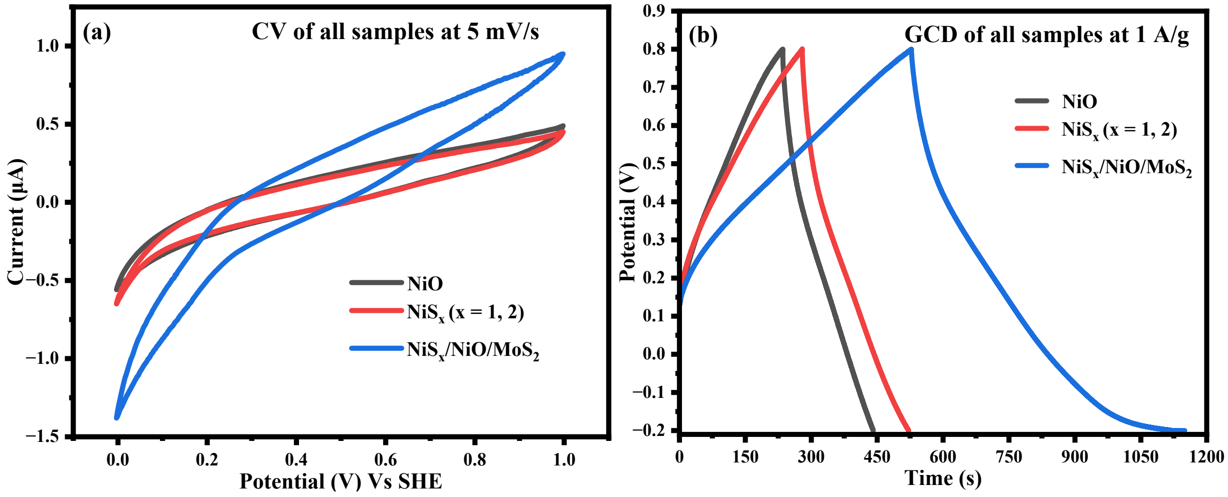

Figure S3: (a) CV curves at 5 mV/s, and (b) GCD plots at 1 A/g of all NiO, NiS_x, and $NiS_x/NiO/MoS_2$ (x = 1, 2) heterostructure.

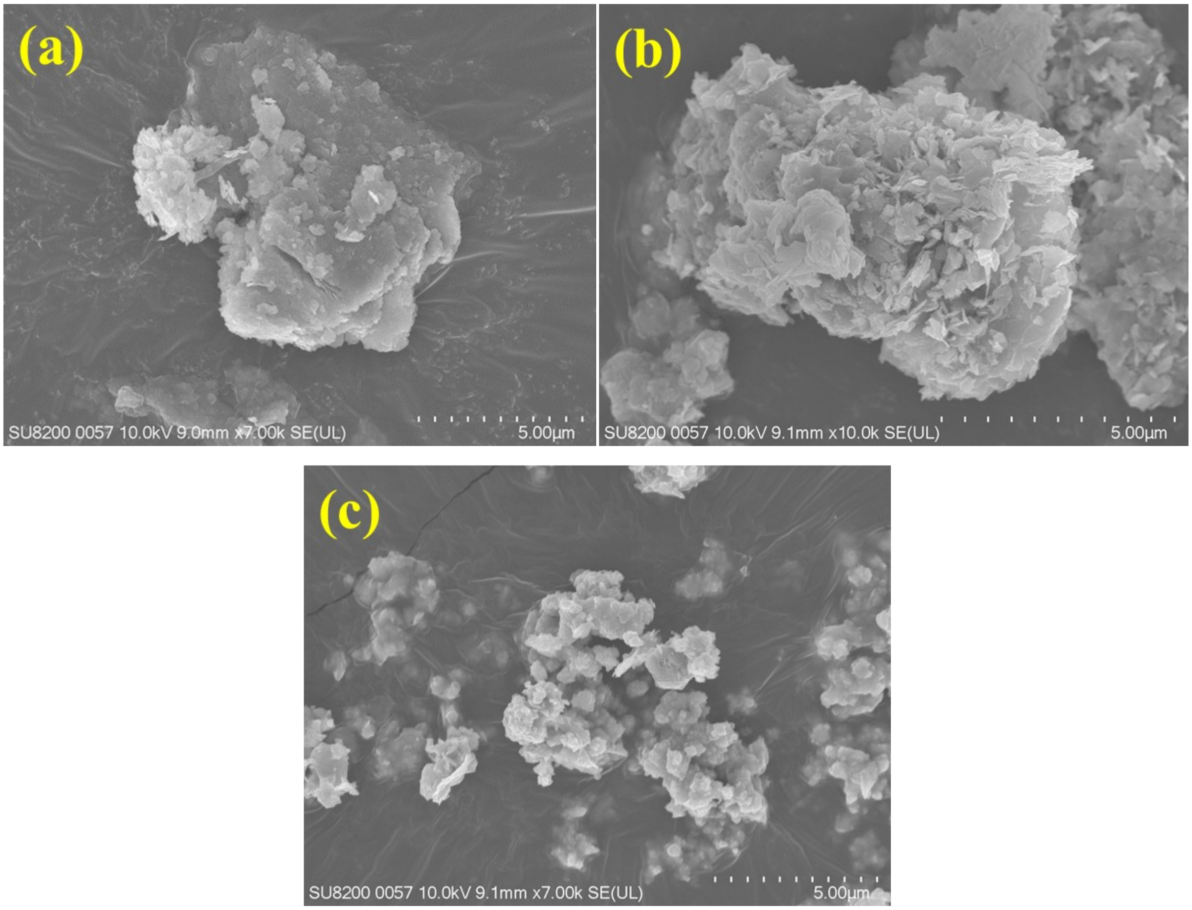

Figure S4: SEM images of (a) NiO, (b) NiS_x, and (c) $NiS_x/NiO/MoS_2$ (x = 1, 2) heterostructure.

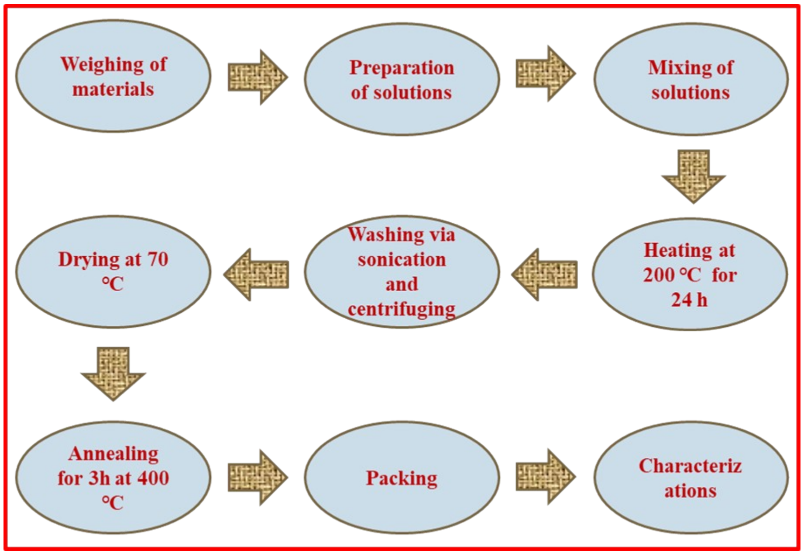

Figure S5: Schematic illustration of the synthesis route of the $NiS_x/NiO/MoS_2$ (x = 1, 2) heterostructure.

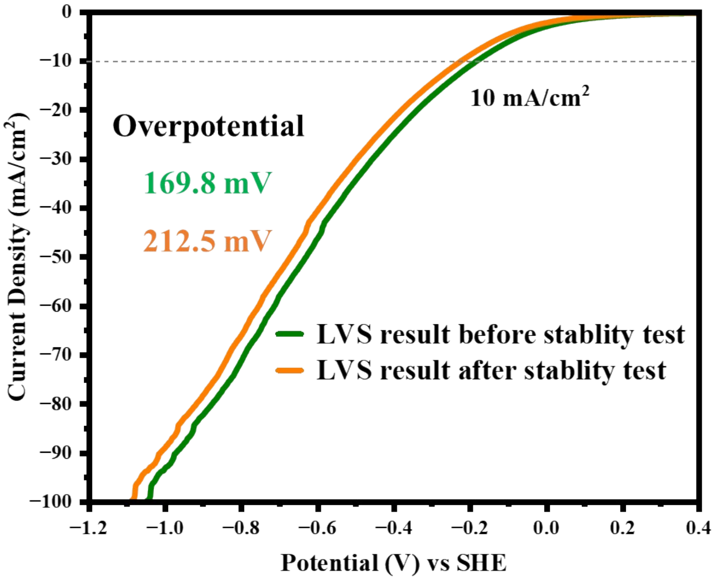

Figure S6: LVS results of the prepared composite before and after the stability test of $NiS_x/NiO/MoS_2$ (x = 1, 2) heterostructure.

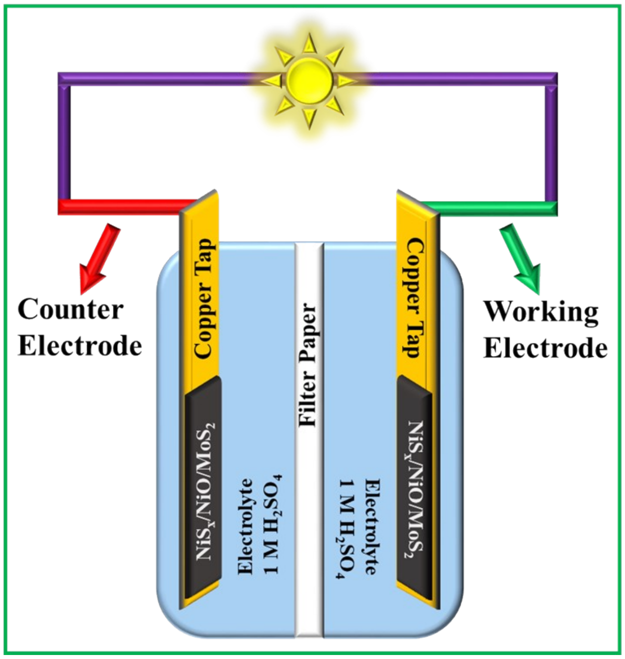

Figure S7: Schematic diagram of the symmetric supercapacitor device

Table S1: A comparative table that highlights the key features of H_2SO_4 and other commonly used hydrogen-based electrolytes

Electrolyte	pH	Proton conductivity (S/cm)	Redox kinetics	Corrosiveness	Common use in
1 M H_2SO_4	~ 0.3	~ 0.08	Fast	Moderate	Supercapacitors, HER/OER
1 M HCl	~ 0.1	~ 0.09	Fast	High	HER, acid corrosion studies
1 M HNO_3	~ 0.3	~ 0.07	Moderate	High	Catalysis, oxidation studies
1 M CH_3COOH	~ 2.4	~ 0.002	Slow	Low	Organic/biochemical systems

Table S2: Comparison of the overpotential, specific capacitance, synthesis method, and performance notes of $NiS_x/NiO/MoS_2$ (x = 1, 2) heterostructure with literature.

Material	Overpotential at 10 mA/cm^2 (mV)	Specific Capacitance (F/g)	Synthesis Method	Performance Notes	Reference
NiS/NiS_2 composite with layered structure	180	95	Hydrothermal	NiS/NiS_2 composite exhibits excellent HER activity and stability	[1]
$NiO/NiS_2/MoS_2$ ternary composite with tunable sulfur vacancies	120	115	Hydrothermal	$NiO/NiS_2/MoS_2$ composite provides high stability and low overpotential	[2]
NiS/MoS_2 composite with MoS_2 edges exposed	150	125	Chemical bath deposition	Enhanced HER efficiency due to the exposed MoS_2 edges	[3]
$NiS_x/NiO/MoS_2$	166	428.2425	Hydrothermal		Our work

References

1. Dhakal, G., Sahoo, S., Sharma, K. P. A Review on the Recent Advancements of Ni-Based Sulfides and Mixed Sulfides for Supercapacitors and Electrocatalysis (Oxygen Evolution Reaction). *Molecules*, *30* (13), 2025.
2. Shah, S. A., Sayyar, R., Xu, L., Sun, H., Khan, I., Guo, J., ... & Ullah, H. In-situ synthesis of NiS_2 nanoparticles/MoS_2 nanosheets hierarchical sphere anchored on reduced graphene oxide for enhanced electrocatalytic hydrogen evolution reaction. *Journal of Colloid and Interface Science*, *624* (18), 2022.
3. Wang, W., Li, L., Tan, S., Wu, K., Zhu, G., Liu, Y., ... & Yang, Y. Preparation of NiS_2//MoS_2 catalysts by two-step hydrothermal method and their enhanced activity for hydrodeoxygenation of p-cresol. *Fuel*, *179* (37) 2016.
